# A *Drosophila* Model of HPV E6-Induced Malignancy Reveals Essential Roles for Magi and the Insulin Receptor

**DOI:** 10.1371/journal.ppat.1005789

**Published:** 2016-08-18

**Authors:** Mojgan Padash Barmchi, Mary Gilbert, Miranda Thomas, Lawrence Banks, Bing Zhang, Vanessa J. Auld

**Affiliations:** 1 Department of Zoology, University of British Columbia, Vancouver, Canada; 2 Department of Biology, University of Oklahoma, Norman, Oklahoma, United States of America; 3 Division of Biological Sciences, University of Missouri, Columbia, Missouri, United States of America; 4 International Centre for Genetic Engineering and Biotechnology, Trieste, Italy; Penn State University School of Medicine, UNITED STATES

## Abstract

Cervical cancer is one of the leading causes of cancer death in women worldwide. The causative agents of cervical cancers, high-risk human papillomaviruses (HPVs), cause cancer through the action of two oncoproteins, E6 and E7. The E6 oncoprotein cooperates with an E3 ubiquitin ligase (UBE3A) to target the p53 tumour suppressor and important polarity and junctional PDZ proteins for proteasomal degradation, activities that are believed to contribute towards malignancy. However, the causative link between degradation of PDZ proteins and E6-mediated malignancy is largely unknown. We have developed an *in vivo* model of HPV E6-mediated cellular transformation using the genetic model organism, *Drosophila melanogaster*. Co-expression of E6 and human UBE3A in wing and eye epithelia results in severe morphological abnormalities. Furthermore, E6, via its PDZ-binding motif and in cooperation with UBE3A, targets a suite of PDZ proteins that are conserved in human and *Drosophila*, including Magi, Dlg and Scribble. Similar to human epithelia, *Drosophila* Magi is a major degradation target. Magi overexpression rescues the cellular abnormalities caused by E6+UBE3A coexpression and this activity of Magi is PDZ domain-dependent. *Drosophila* p53 was not targeted by E6+UBE3A, and E6+UBE3A activity alone is not sufficient to induce tumorigenesis, which only occurs when E6+UBE3A are expressed in conjunction with activated/oncogenic forms of Ras or Notch. Finally, through a genetic screen we have identified the insulin receptor signaling pathway as being required for E6+UBE3A induced hyperplasia. Our results suggest a highly conserved mechanism of HPV E6 mediated cellular transformation, and establish a powerful genetic model to identify and understand the cellular mechanisms that underlie HPV E6-induced malignancy.

## Author Summary

Human papillomaviruses (HPV) are the causative agents of cervical cancer, one of the leading causes of cancer death in women worldwide. The E6 oncoprotein encoded by HPV has been implicated in the progression of primary tumors to metastatic disease and we have developed a new model in the fruit fly (*Drosophila melanogaster*) to study the cellular effects of E6. The E6 protein recruits an E3 ubiquitin ligase (UBE3A) to induce the degradation of a number of cellular proteins, including members of the MAGUK family of scaffolding proteins that control the structure and polarity of epithelial cells: Dlg, Scribble and Magi. Expression of E6 and human UBE3A in the wing and eye of *Drosophila* disrupted these tissues. Similar to human cells we found that *Drosophila* Magi was a major E6 degradation target and that overexpression of Magi rescued the tissue disruption. However, *Drosophila* p53 was not degraded by E6/UBE3A, making our fly model potentially useful for studying the p53-independent activities of the E6+UBE3A complex. When we paired E6 expression with oncogenic proteins, including activated Ras, we observed that epithelia were transformed into mesechymal-like cells that left the epithelium and spread through the body. As a test of the potential of our system, we carried out a pilot genetic screen and identified the insulin receptor as a strong modulator of the E6-mediated disruption of *Drosophila* tissues. Therefore, we have developed a new system and approach to help us better understand the mechanisms that underlie how HPV infection leads to cell transformation and cancer.

## Introduction

Cervical cancer is the fourth leading cause of cancer death in women worldwide with ~500,000 new cases of cervical cancer annually and ~250,000 deaths worldwide. The main causative agents of cervical cancer are the high-risk human papillomaviruses (HPVs). HPVs can induce hyperproliferative lesions in epithelia and are responsible for >90% of cervical and anal cancer, and more than 50% of vaginal, vulvar, penile and oropharyngeal cancers as well as a significant number of head and neck squamous cell carcinomas [1–4]. Although HPV vaccines are now available, it is still very important to understand the mechanism of HPV-induced tumorigenesis, given the 20 years or so lag between infection and cancer development and the low rates of vaccine uptake in many regions [5–7]

The HPV oncogenes, E6 and E7, are key to the cell transformation that underlies HPV-mediated cancer. Multiple studies have shown that E6 and E7 work cooperatively to induce carcinogenesis [8,9]. E7 is critical for early stages of tumor formation, causing benign tumors and targeting Rb, whereas E6 is thought to play an important role during the later stages of tumor progression [10–14]. E6 inactivates a range of targets including the tumor suppressor protein p53 and important polarity regulators including hDlg1, Scribble/Vartul and MAGI-1, all of which are directed for ubiquitin-mediated proteasomal degradation [14–19]. E6 directs the degradation of many of its substrates through recruitment of an E3 ubiquitin ligase, UBE3A/E6AP, with which it forms a stable complex and redirects its activity towards the different E6 target proteins [20,21]. Multiple functions of E6, including interaction with UBE3A, p53 and PDZ domain-containing substrates, appear to be required for its ability to bring about cell transformation and to contribute towards malignancy in animal models.

The PDZ binding motif (PBM) of E6 is almost exclusively found in the high-risk HPV types, and is essential for HPV-mediated malignancy and hyperplasia [22]. However, a major question remaining is how E6-mediated degradation of PDZ proteins leads to cellular transformation and malignancy within a living animal. Transgenic mice models of HPV 16 and 18 E6 have contributed to our understanding of HPV-mediated tumorigenesis but the underlying cellular mechanisms and the hierarchy in importance of the PDZ target proteins have not been elucidated.

*Drosophila melanogaster* has been widely and successfully used as a powerful genetic model organism to study human diseases, including cancer, owing to the strong conservation of genes and signaling pathways between *Drosophila* and human. Many tumor suppressor genes, including Dlg, Scribble, and Lgl, as well as oncogenic signaling pathways such as Notch, were first identified in *Drosophila* [23–27]. Many epithelial derived tumors have been modeled in flies. For example, oncogenic Ras or Notch paired with loss of function mutations in Scribble result in the formation of metastatic tumors in *Drosophila* that share many characteristics with human tumors [28–31].

In this study we have developed an *in vivo* model of HPV E6-mediated cellular transformation using *Drosophila*. In this model the levels of E6 expression are high and reflect the higher levels of E6 expression seen during the later stages of malignant progression. We show that coexpression of HPV E6 and human UBE3A/E6AP in the wing and eye results in severe morphological defects, whereas E6 or UBE3A expression alone results in none. We find that E6, in cooperation with UBE3A, targets PDZ proteins in a PBM-dependent manner, and these targets include Magi, Dlg and Scribble with Magi being a major degradation target. In contrast, *Drosophila* p53 was not degraded by E6+UBE3A. In addition to the loss of PDZ scaffolding proteins, E6+UBE3A expression in epithelia led to apoptosis paired with delamination. Importantly, Magi overexpression rescued the cellular abnormalities caused by E6+UBE3A and the Magi PDZ domains were necessary for this rescue. E6+UBE3A activity was not sufficient to induce tumorigenesis, but did result in malignancy when co-expressed with either an activated form of oncogenic Ras or Notch. These cells had the hallmarks of epithelial-mesenchymal transition (EMT) including morphological changes paired with elevated MMP1 and aPKC expression. To identify signaling pathways that modulate the E6+UBE3A effects, we conducted a genetic screen and found that E6+UBE3A interacted with the insulin receptor. Overall, our results establish the first *Drosophila* model to study the HPV E6-mediated cellular transformation and malignancy and suggest a high degree of conservation on the mechanism of HPV E6 mediated cellular transformation.

## Materials and Methods

### Fly strains and genetics

The following *Drosophila* strains were used, UAS-Magi::Cherry[32], UAS-Dlg::GFP [33], UAS-MagiΔPDZ and UAS-MagiΔWW from [34], UAS-hUBE3A from [35], Scrib::GFP [36], UAS-Scrib::GFP [37]. UAS-p35, UAS-mCD8::GFP, UAS-InR, UAS-InR DN, UAS-ArmS10, EGFR DN, UAS-rl^Sem^, UAS-Yki::GFP, UAS-Notch Intra, UAS-Bsk DN, UAS-Ras85D.V12, P53::GFP, UAS-P53 H159N, UAS-Debcl, *buffy* ^*H37*^, *Debcl* ^*E26*^, apterous-Gal4, GMR-Gal4, Gal80^ts^ and all the transgenes and mutants used in the Table 1 were from the Bloomington *Drosophila* Stock Center.

**Table 1:** Signaling pathway screen to identify E6+UBE3A interacting genes.

Genes tested	Allele type	Signaling Pathway	Effect on E6+UBE3A
UAS-InR	Gain of function	Insulin signaling	Enhanced proliferation
UAS-InR DN	Loss of function	Insulin signaling	Enhanced necrosis
*Akt[04226]/+*	Loss of function	PI3K signaling	No effect
	Strong hypomorph		
UAS-Rolled Act	Gain of function	MAPK signaling	No effect
UAS-EGFR DN	Loss of function	EGFR signaling	No effect
UAS-Ras85D DN	Loss of function	Ras signaling	No effect
UAS-Arm S10	Gain of function	Wnt signaling	No effect
UAS-Yki::GFP	Gain of function	Hippo signaling	No effect
*hh[AC]/+*	Loss of function	Hedgehog signaling	No effect
	amorphic		
*wit[A12]/+*	Loss of function	BMP signaling	No effect
	Strong hypomorph		
UAS-Bsk DN	Loss of function	JNK signaling	No effect
UAS-Dronc DN	Loss of function	Apoptosis—JNK	No effect
Eiger-RNAi	Loss of function	Apoptosis–JNK	No effect
*Tak1[2527]/+*	Loss of function	JNK signaling	No effect
*buffy[H37]/+*	Loss of function	Apoptosis	No effect
*DebclE26[]/+*	Loss of function	Apoptosis	No effect
UAS-Debcl	Gain of function	Apoptosis	No effect
UAS-P53 H159N	Loss of function	Apoptosis	No effect
*hop[2]/+*	Loss of function	JAK/STAT signaling	No effect
	amorphic		
*Stat92E[06346]/+*	Loss of function	JAK/STAT signaling	No effect
	amorphic		

### Generation of UAS-E6 transgenic lines

HPV18-E6WT and HPV18-E6V158A are described in Thomas et al (2005) [18]. Each cDNA was re-derived by PCR using oligos: 5’-accggtATGGCGCGCTTTGAGGATC-3’ for the 5 prime side of both clones and either 5’-ctcgagTTATACTTGTGTTTCTCTGC-3’ for the wild type 3 prime end, or 5’-ctcgagTTATAGTTGTGTTTCTCTGC-3’ for the Val-to-Ala mutant 3’ end. The underlined codons correspond to the mutated amino acid. All products were subcloned into pGEM-T (Promega) and verified by sequence analysis. Each product was subcloned into pBluescript(KS+) (Stratagene) using Not I and Sac II restriction sites, then transferred into pUASTattB [38] modified to contain either Myc, HA, or Flag epitope tags 5’ to the multiple cloning site, using Age I and Xho I restriction sites. Injections and integrase-mediated insertion into *w-; P(CaryP)attP2* was carried out by Rainbow Transgenic Flies, Inc (Camarillo, CA, USA).

### Immunohistochemistry

For immunolabeling, wing discs from wandering third instar larvae and pupal eyes 42 hrs after puparium formation were dissected in PBS and fixed in 4% formaldehyde. Fixed tissues were washed three times in PBS solution containing 0.1% Triton-X-100 and blocked in 5% normal goat serum for 1 hour before incubation with primary antibodies. The primary antibodies used in this study were rabbit anti-Magi 1:200 [39], rabbit anti-Baz 1:1000 [40], mouse anti-Dlg 1:50 (4F3, Developmental Studies Hybridoma Bank), rat anti-DE-cadherin DCAD2 1:50 (Developmental Studies Hybridoma Bank), mouse anti-Flag 1:300 (M2, Sigma), rabbit anti-cleaved Cas3 1:300 (Cell Signaling), rabbit anti-Myc 1:200 (Abcam) and rabbit anti-HA 1:200 (Abcam) [41], rat anti-Magi 1:300 [42], mouse anti-Arm 1:50 (Developmental Studies Hybridoma Bank), mouse anti-MMP1 1:100 (Developmental Studies Hybridoma Bank), rabbit anti-aPKC zeta C20 1:1000 (Santa Cruz Biotechnology). The appropriate secondary antibodies were conjugated Alexa488, Alexa594, and Alexa 647 (Invitrogen).

Images were collected using a Leica SP8 Scanning multiphoton confocal microscope, processed in ImageJ. Figures were assembled using Adobe Photoshop. Low magnification images were collected on a Zeiss Axioskop with a 5X NA 0.50 air lens and AxioVision software. For quantification of protein levels, the fluorescence intensity at the plasma membrane was measured for a standardized ROI in both the overexpression and non-overexpression sides, using the “Find Edges” function of ImageJ followed by threshold adjustments and measurement of intensity. The data were then transferred to Excel for further analysis and plot creation. For each experiment five wing imaginal discs were analyzed and an unpaired t-test was used for statistical analysis.

For quantification of the degree of rescue by Magi::cherry, MagiΔWW, MagiΔPDZ, Dlg::GFP, Scrib::GFP, flies that fulfilled all the following criteria were considered rescued: 1.full wings, 2. only small melanized blisters, 3. fully differentiated wing veins and margins. Flies with rescued wing phenotypes were counted and divided by the total number of flies assayed and percentage was calculated.

### Scanning Electron Microscope of fly eyes

For SEM of fly eyes heads were fixed in 4% glutaraldehyde in PBS, washed three times to remove the fixative and subsequently dehydrated in an ethanol series. After dehydration samples were critical point dried and mounted on SEM stubs followed by sputter coating with a thin layer of AuPd. Samples were imaged using a Zeiss Neon high-resolution scanning electron microscope.

### *In vitro* degradation assays

These assays were performed as described previously [43]. Briefly, *Drosophila* Magi and mammalian MAGI-1 proteins were transcribed and translated *in vitro*, using the TnT kit (Promega), and radiolabelled with [35S]-Methionine (GE Healthcare). They were incubated at 30°C for the indicated times, with or without the addition of similarly translated HPV-16 and HPV-18 E6 proteins. The remaining proteins were detected by SDS-PAGE and autoradiography.

## Results

### Establishing a fly model of HPV E6 and human UBE3A/E6AP

**Fig 1:**
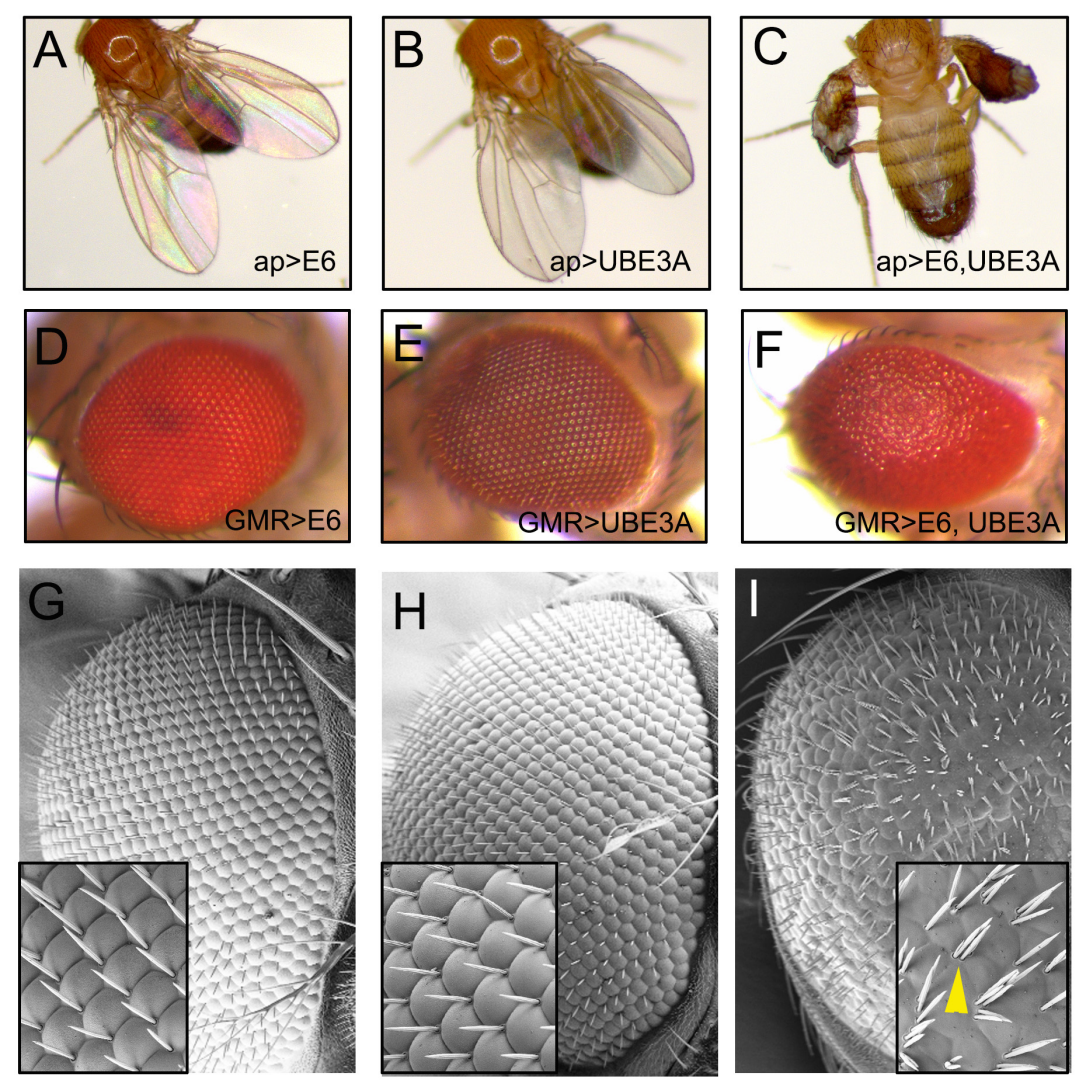
E6 in cooperation with UBE3A causes severe wing and eye abnormalities. **(A-C)** Transgenes were driven with apterous-Gal4 in the wing imaginal disc. Expression of HPV18 E6 **(A)** or human UBE3A **(B)** alone had no effect on adult wing morphology. **(C)** Co-expression of E6 and UBE3A resulted in severe abnormalities. Wings are blistered and full of melanized fluid, with indistinct structure and lacking veins. **(D-I)** Transgenes were driven with GMR-Gal4 in the eye imaginal disc. Expression of E6 **(D)** or UBE3A **(E)** alone had no effect on adult eye morphology. **(F)** Coexpression of E6 and UBE3A in the eye generated a disorganized “rough eye” phenotype. **(G-I)** Scanning electron microscopy of eyes from **(D-F)**. E6 **(G)** or UBE3A **(H)** alone had no effect on ommatidia organization or bristles. **(I)** When E6 and UBE3A are co-expressed ommatidia were fused with increased bristles (arrowhead).

While transgenic mouse models of HPV E6 have significantly contributed to the understanding of HPV-mediated tumorigenesis, the role of the HPV-E6 PDZ targets *in vivo* has not been clearly established. As there are no other *in vivo* models of HPV E6 at present, and given the wide array of genetic tools and techniques available in fruit flies, we felt that developing a fly E6 model would provide the ability to further dissect the molecular pathways and identify novel partners involved in HPV E6-mediated cellular transformation. To do this, we expressed genes encoding the human HPV18 E6 tagged with Myc in two separate epithelia using the Gal4-UAS high-expression system [44]. When we expressed E6 in the wing or eye epithelia, using the *apterous*-GAL4 and GMR-GAL4, respectively, we did not detect any abnormalities within these tissues (Fig 1A, 1B, 1D, 1E, 1G and 1H). E6 requires the human E3 ubiquitin ligase (UBE3A/E6AP) for many of its functions. While *Drosophila* has a UBE3A homologue it was possible that HPV E6 was unable to activate or interact with the *Drosophila* UBE3A. This suggests that the human UBE3A has a specific interaction with E6 or a function that is not conserved in the *Drosophila* UBE3A protein. The former is likely as the LXXLL motif necessary for E6 binding to human UBE3A [45] is absent from *Drosophila* UBE3A (this study). In support of this we found that expression of E6 together with a previously established transgene that expresses human UBE3A [35] resulted in severe abnormalities in wing and eye epithelia (Fig 1C, 1F and 1I). These abnormalities included smaller and blistered wings that were full of melanized tissue and were held out. Similarly, co-expression of E6 and human UBE3A in the eye resulted in rough eyes with disorganized and fused ommatidia as well as increase in number of bristles (Fig 1F and 1I). These abnormalities were specific to co-expression of E6 and UBE3A and were 100% penetrant, whilst neither E6 nor UBE3A expression alone resulted in any defects. These results show that expression of E6+UBE3A is deleterious in *Drosophila* epithelia, suggesting a conservation of cellular targets downstream of the E6+UBE3A complex.

### E6 degrades *Drosophila* PDZ domain proteins

**Fig 2:**
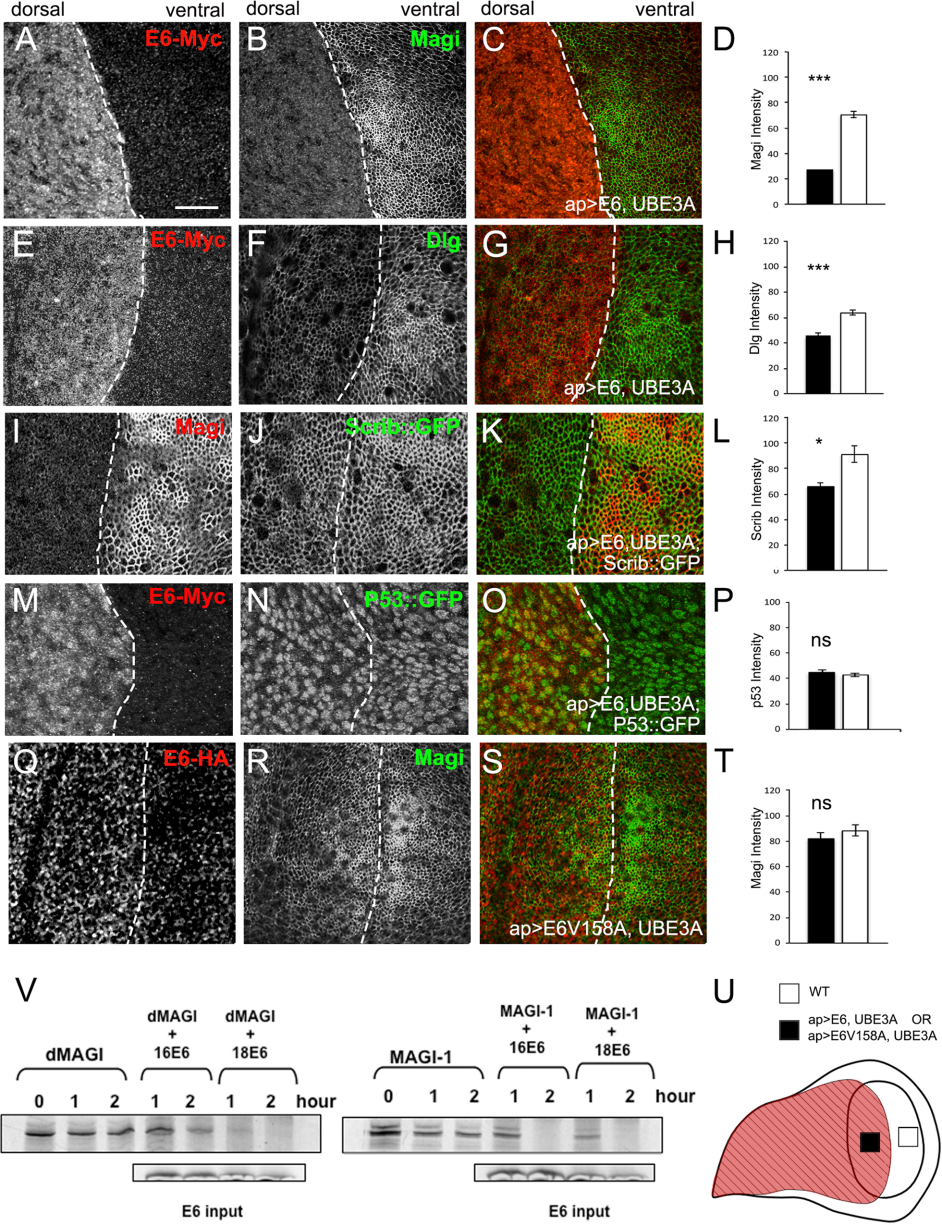
E6-mediated degradation of PDZ domain proteins is conserved in *Drosophila*. **(A-T)** apterous-Gal4 was used to express transgenes in the dorsal compartment of wing imaginal discs. In all images dorsal is to the left and ventral is to the right and dashed lines indicate the boundary between the two compartments. Single Z slices are shown. **(A-P)** E6 and UBE3A co-expression results in a severe loss of Magi **(A-C)**, a less severe reduction of Dlg **(E-G)** and slightly reduced level of Scrib **(I-K)**. Immunolabeling was quantified and the reductions were significant compared with the control dorsal side for Magi (p<0.001, n = 5 discs) **(D)**, Dlg (p<0.001, n = 5 discs) **(H)** and Scrib (p<0.05, n = 5 discs) **(L)**. **(M-O)** Co-expression of E6 and UBE3A had no effect on the expression level or localization of P53. **(P)** Quantification of the level of P53 found no significant change (n = 5 discs). **(Q-S)** Coexpression of an E6 transgene lacking the PDZ binding motif (E6V158A) and UBE3A did not effect the levels or localization of Magi. Quantification of the level of Magi found no significant change (n = 5 discs) **(T)**. **(U)** The apterous expression pattern (red) is diagrammed on the wing imaginal disc. Quantification of immunolabeling was from set standardized areas on the apterous side (black square) compared to the wild type side (white square). **(V)** *Drosophila* Magi and mammalian MAGI-1 are both susceptible to degradation induced by the high-risk cancer-causing HPV 16 and 18 E6 proteins. *In vitro* degradation assay reveals that HPV16E6 and HPV18E6 are able to degrade radiolabeled *Drosophila* Magi to an extent nearly equal to human MAGI. *** p<0.001, * p<0.05, ns—not statistically significant. Error bars indicate SEM. Scale bars indicate 10μm.

The wing and eye phenotypes prompted us to ask whether any of the known targets of E6 in human cells are also targeted in *Drosophila* epithelia. E6 targets PDZ domain proteins of the polarity and junctional network including MAGI-1, hDlg1 and Scribble/Vartul for ubiquitin-mediated proteasomal degradation [14–19], an activity that requires an intact PBM at the extreme C terminus of E6 [22]. We focused on the wing imaginal disc using the *apterous*-GAL4 driver to drive expression in the dorsal half of the wing disc to allow for direct comparison of protein levels in the presence and absence of E6+UBE3A. We focused our attention on the *Drosophila* homologues of Magi, Discs-large (Dlg) and Scribble (Scrib) and monitored the protein levels on the dorsal (expressing E6+UBE3A) versus ventral (lacking E6+UBE3A) side of the wing imaginal disc (Fig 2). We found that E6+UBE3A co-expression led to a significant loss of Magi from the adherens junction domain (Fig 2A–2D), a modest reduction in the levels of Dlg (Fig 2E–2H), and a weak reduction in the levels of Scrib (Fig 2I–2L) at their respective locations in the septate junction. In order to determine if all potential PDZ proteins were effectively targeted by E6, we examined the levels and localization of two other PDZ proteins that are known to be in cell junctions, Bazooka (Par-3) or Par-6, and found no effect on the levels or the localization of these PDZ protein (S1A–S1H Fig). These results indicate that E6 binding and degradation of a select group of PDZ proteins is specific. Similarly, there were no changes in the level or localization of E-cadherin at the adherens junction (S1I–S1L Fig). Expression of E6 or UBE3A alone did not result in any changes in the levels or localization of Magi, Dlg or Scribble (S1M–S1X Fig), confirming that alterations to the PDZ proteins are a result of the E6 and UBE3A complex. Our results are consistent with previous results from human cervical cancer cells in which MAGI-1, hDlg-1 and hScrib are targeted for degradation by E6. In contrast, *Drosophila* p53 was not targeted for degradation by E6+UBE3A (Fig 2M–2P), which is consistent with evolutionary differences between human and fly p53. Our results suggest that *Drosophila* Magi is particularly susceptible to E6 targeting. This is consistent with studies performed in mammalian cells, where MAGI-1 is one of the most strongly bound E6 PDZ targets and is also very efficiently degraded [15,43,46,47]. This suggests that the mechanism of E6 targeting these PDZ cell polarity-regulating proteins is conserved between humans and *Drosophila*.

As Magi appears to be a major target of HPV E6+UBE3A in *Drosophila* we were interested in directly comparing the ability of E6 to degrade human MAGI-1 and *Drosophila* Magi *in vitro* (Fig 2V). *Drosophila* Magi and human MAGI-1 were transcribed and translated *in vitro*, using the TnT rabbit reticulocyte lysate system, which includes functional UBE3A/E6AP [48]. These proteins were then incubated with similarly translated HPV-16 or HPV-18 E6 and the remaining protein analysed by SDS PAGE and autoradiography. As can be seen from Fig 2V, *Drosophila* Magi and mammalian MAGI-1 are almost equivalently susceptible to degradation induced by the high-risk cancer-causing HPV 16 and 18 E6 proteins. These findings highlight the high degree of evolutionary conservation in this particular HPV E6 target, and provide the molecular basis for the results obtained *in vivo*.

### Degradation of PDZ proteins requires the E6 PBM

HPV E6-mediated degradation of PDZ proteins in human epithelial cells requires an intact PBM at the C-terminus of the E6 protein [22]. We next tested whether the loss or reduction of PDZ proteins in the *Drosophila* epithelial cells was also dependent upon an intact E6 PBM. To do this, we generated a transgene expressing E6 with a point mutation that disrupts the E6 PBM (E6V158A) and prevents E6 binding to PDZ domain proteins [43]. When co-expressed with UBE3A, the E6V158A mutant did not trigger a loss of Magi (Fig 2Q–2T), Dlg or Scrib (S1Y-S1Z’ Fig). These results confirm that E6 targeting of *Drosophila* Magi requires an intact E6 PBM.

### Co-expression of HPV E6 and E6AP causes cellular abnormalities in the eye and wing epithelia

**Fig 3:**
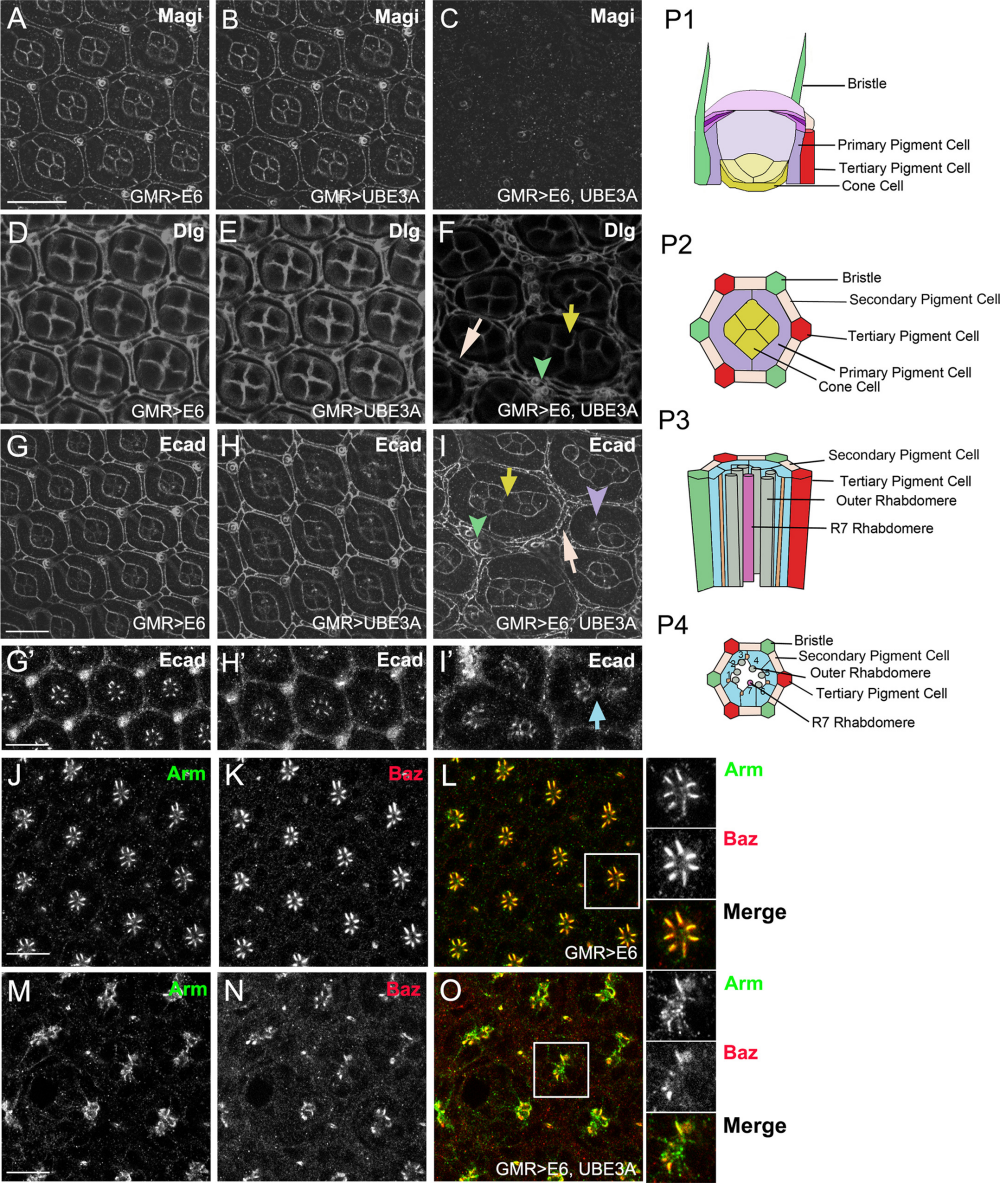
Co-expression of E6 and UBE3A in the eye disrupts the cellular integrity and structure. Expression of transgenes in the eye imaginal disc were driven with GMR-Gal4. **(A-C)** Expression of E6 **(A)** or UBE3A **(B)** alone had no effect on Magi. **(C)** Coexpression of E6 and UBE3A resulted in a loss of Magi. **(D-F)** Expression of E6 **(D)** or UBE3A **(E)** alone had no effect on Dlg. **(F)** Coexpression of E6 and UBE3A reduced the level of Dlg. **(G-I)** Immunolabeling with Ecad showed no effect on ommatidial organization with expression of E6 **(G)** or UBE3A alone **(H)**. **(I)** Ecad immunolabeling of eyes expressing E6+UBE3A indicated disrupted cellular morphology and integrity. **(F, I)** Multiple phenotypes were observed with E6+UBE3A expression including fused ommatidia observed with increased primary cone cells (yellow arrow). The number of secondary pigment cells (white arrow), primary pigment cells (purple arrowhead) and bristle cells (green arrowhead) were increased and the organization of ommatidia is also perturbed. **(G’-I’)** En-face view from a deeper plane of the eye at the level of the rhabdomere and photoreceptor cells. Rhabdomeres from neighboring ommatidia are fused **(I**, blue arrow) compared to H’ and G’. **(J-L)** Expression of E6 alone had no effect on the polarity protein Baz and junctional Armadillo (Arm), which normally localize to the zona adherens in photoreceptors and appear yellow in L and enlarged boxed area (inserts). **(M-O)** Coexpression of E6 and UBE3A disrupted the integrity of junctional complexes, as Baz and Arm were both mislocalized as shown by reduced amount of yellow overlap in O and enlarged boxed area (insets). **(P1-4)** Diagram of an ommatidium in the eye with side views (P1, P3) and corresponding cross sections (P2, P4) from the outer most level and the inner rhadomere/photoreceptor level. (P1, P2) The upper part of ommatidium is a composition of 4 cone cells (yellow) in the center of the ommatidium surrounded by two primary pigment cells (purple), 6 secondary pigment cells (beige), 3 tertiary pigment cells (red) and 3 bristle cells (green) that generate the eye bristles. P2 represents the cells shown in A-I. **(P3-P4)** A more interior level of the eye at the level of the eight photoreceptor cells (blue) and their rhabdomeres (gray), which are the apical domain of photoreceptors. P4 corresponds to panels G’-O. Scale bars indicate10μm. Insets are digitally magnified 200%.

The columnar epithelia of the *Drosophila* imaginal disc are ideal for studying cellular transformation and cellular signaling pathways, and have been used as *in vivo* models for detailed analysis of cellular and molecular mechanisms underlying cell polarity defects and cancers [29,30,49]. The eye disc in particular has been an excellent model to study the molecular and signaling mechanisms that underlie cell transformation and progression to cancer. When E6+UBE3A was co-expressed in the eye the earliest cellular phenotypes were observed during pupal stages of development. We observed a loss of Magi (Fig 3C) and a reduction in Dlg in the eye imaginal disc in the presence of E6+UB3EA (Fig 3F): this mirrors what we observed in the wing imaginal disc where Magi levels were extensively reduced while Dlg was less so. E6+UBE3A co-expression in the eye resulted in severe tissue abnormalities. In the normal eye ommatidia are arranged in a hexagonal array to produce a stereotyped pattern. Each ommatidium consists of eight photoreceptor cells, four cone cells, three types of pigment cells (primary, secondary and tertiary) and bristle cells that form the bristles in the adult eye [50](Fig 3P). In eyes coexpressing E6 and UBE3A the overall structure of the eye and the organization of ommatidia was disrupted. Frequently, neighboring ommatidia were fused (70% of ommatidia; n = 10 discs) and there was an increase in the number of cone cells, primary, secondary and tertiary pigment cells, as well as bristle cells (Fig 3F and 3I). These cellular defects were evident in the adult eye with an increase in the number of bristles and the rough or glossy eye phenotype (Fig 1G–1I). Immunolabeling for junctional and polarity markers, Ecad, beta-catenin (*Drosophila* Armadillo, Arm) and Par3 (*Drosophila* Bazooka, Baz), revealed that the organization of photoreceptor cells within each ommatiduim was perturbed and that junctional as well as polarity proteins were mislocalized (Fig 3G–3O). The displacement of these markers suggests that E6 disrupts the cell polarity and junctional integrity of photoreceptors in ommatidia.

**Fig 4:**
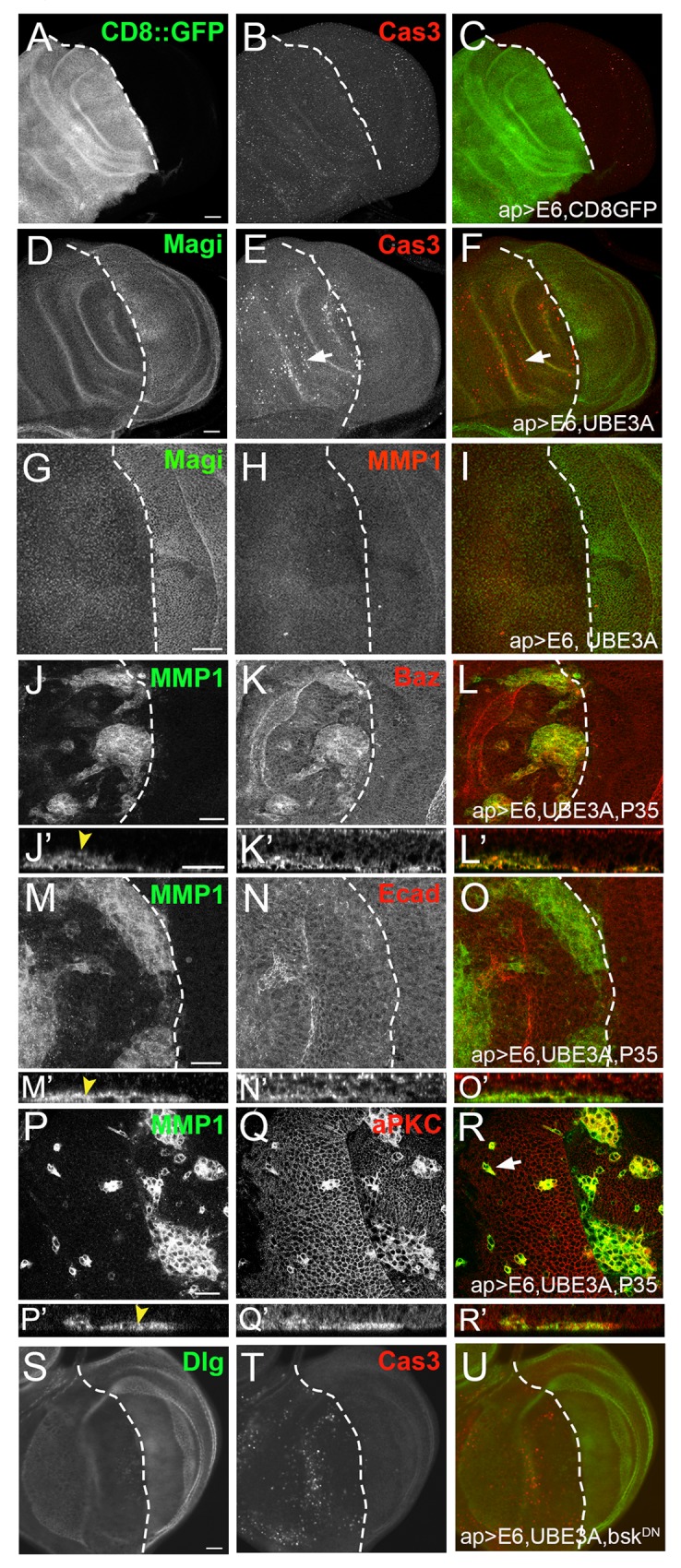
Co-expression of E6 and E6AP induces cellular transformation that is dependent on inhibition of apoptosis. All transgenes were driven with apterous-Gal4 in dorsal compartment of wing discs. In all images dorsal is to the left and ventral is to the right and the boundary indicated by dashed lines. **(A-C)** Expression of E6 alone did not result in apoptosis as detected using an antibody to cleaved caspase 3 (Cas3). **(D-F)** Co-expression of E6 and UBE3A causes cell death marked by the increased immunolabeling for cleaved caspase 3 (arrow). **(G-I)** Coexpression of E6 and UBE3A did not increase MMP1 expression. **(J-L)** Blocking cell death with p35 in cells expressing E6 and UBE3A resulted in cell clusters expressing high levels of MMP1and Baz. **(J’-L’)** Side projections indicating the delaminated cell clusters were on the basal side of the epithelium (yellow arrowhead). **(M-O)** MMP1-expressing cell clusters in E6+UBE3A+p35-expressing epithelia showed no increase in junction protein Ecad. **(M’-O’)** Side projection of M-O showing delaminated cell clusters on the basal side of the disk (yellow arrowhead). **(P-R)** Cell clusters within the apterous domain expressing E6+UBE3A+p35 had elevated MMP1 and aPKC levels. **(P’-R’)** Side projection of P-R showing delaminated cell clusters on the basal side of the disc strongly immunolabeled with MMP1 and aPKC (yellow arrowhead). **(S-U)** Dominant negative *Drosophila* JNK, Bsk (bskDN), blockade of JNK signaling did not suppress the cell death or elevated immunolabeling for Cas3 resulting from coexpression of E6 and UBE3A. Scale bars in A-F and S-U are 20μm and 10μm in all other panels.

To determine whether the effects of E6+UBE3A were dosage dependent, we increased the expression of E6 and UBE3A by increasing the temperature to 29°C to increase the efficacy of Gal4 [51]. At 29°C, E6+UBE3A triggered extensive apoptosis on the apterous side compared with the control wildtype side, as detected by an antibody to cleaved-Caspase 3 (Fig 4D–4F). Normally, expression of the baculovirus protein p35 blocks apoptosis, leading to compensatory proliferation or apoptosis-induced proliferation [52–54]. Blocking apoptosis with p35 [41] did not result in overgrowth when co-expressed with E6+UBE3A, but instead resulted in clusters of cells that expressed high levels of the polarity proteins, Baz and aPKC (Fig 4J–4L) in all discs observed (n = 20). Increased expression of vertebrate Par3 (Baz) and aPKC are associated with tumorigenesis [55] and progressive stages of cancer and EMT [56–58]. A common marker of transformation and EMT is the increased expression of matrix metalloproteinase 1 (MMP1) [59,60] and we observed that all cell clusters expressed high levels of MMP1 (Fig 4J–4R), suggesting that these cells were undergoing EMT. The cell clusters were not within the columnar epithelia, but were found under the epithelium at the basal side (Fig 4J’–4R’; arrowheads). We also observed individual cells expressing high levels of MMP1 and polarity proteins away from the cell clusters within the basal side (Fig 4P–4R, arrow). Conversely, when we examined the level and localization of the adherens junction protein Ecad, we did not detect any increase in the delaminated cell cluster. These results suggest that HPV E6, in cooperation with UBE3A, triggers apoptosis and subsequent cell delamination when apoptosis is blocked.

Consistent with previous studies from vertebrates, E6+UBE3A expression alone was insufficient to induce cellular transformation and only when it is paired with processes that block apoptosis is cell transformation observed. As c-Jun N-terminal kinase (JNK) is one of the main signaling pathways triggering apoptosis and MMP1 expression, we expressed a dominant negative form of *Drosophila* JNK (bsk^DN^) to block JNK signaling in wing discs co-expressing E6+UBE3A. Blocking JNK signaling did not suppress the E6+UBE3A-mediated cell death (Fig 4S–4U), suggesting that the E6+UBE3A mediated apoptosis did not involve the JNK signaling pathway and was driven through another cellular pathway.

### Overexpression of dMagi rescues the abnormalities caused by HPV E6+UBE3A

**Fig 5:**
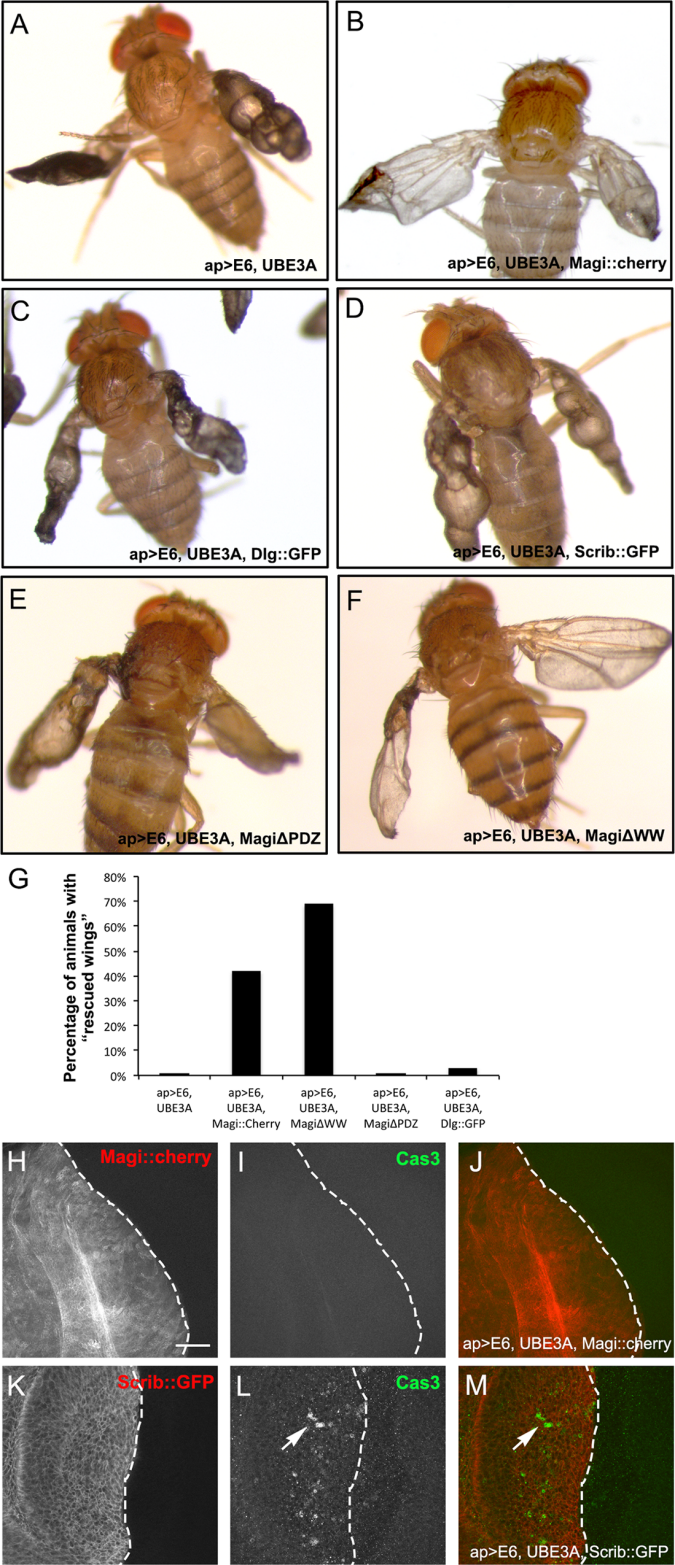
Overexpression of *Drosophila* Magi rescues the HPV E6 + UBE3A wing phenotypes. Transgenes were expressed using apterous-Gal4 driver in the dorsal compartment of the wing disc, affecting the hinge region joining the wing to thorax and one surface of the adult wing. **(A)** Coexpression of E6 and UBE3A results in adult wings that are entirely blistered, full of melanized tissues and lacking wing veins. **(B)** Overexpression of Magi::Cherry with E6+UBE3A suppressed the wing blister and melanization phenotype. Wings were fully formed and all the wing veins were present. Rescue was partial with some blisters still detected. **(C,D)** Overexpression of Dlg::GFP **(C)** or Scrib::GFP **(D)** in wing discs expressing E6+UBE3A did not suppress any of the E6+UBE3A-mediated phenotypes. **(E,F)** Overexpression of MagiΔPDZ **(E)** did not suppress while overexpression of MagiΔWW **(F)** in the wing discs expressing E6+UBE3A suppressed both the wing blister and melanization phenotypes. Wings were fully formed and all the wing veins were present. **(G)** The percentage of animals with rescued wings, as outlined in Material and Methods, were quantified (n = 100 for each genotype). **(H-M)** Analysis of third instar wing discs. In all panels dorsal is to the left and ventral is to the right. Dashed lines indicate the boundary between the dorsal and ventral compartments. **(H-J)** Overexpression of Magi (Magi::cherry) in wing discs blocked the apoptosis triggered by E6+UBE3A expression, as shown by the lack of activated Cas3 immunolabeling (Cas3). **(K-M)** Overexpression of Scrib::GFP in wing discs expressing E6+UBE3A did not block apoptosis, as shown by activated Cas3 immunolabeling (arrow). Scale bar indicates 10μm.

As Magi was strongly reduced by E6+UBE3A expression, we next asked whether Magi overexpression could suppress and rescue the defects caused by E6+UBE3A in the wing epithelia. We found that co-overexpression of a Cherry-tagged form of *Drosophila* Magi (Magi::Cherry) and E6+UBE3A partially rescued the adult wing phenotypes (Fig 5B). Specifically, there was less melanized tissue, a reduced degree of blistering and the flies exhibited full wings with fully differentiated veins and wing margins. We quantified the degree of rescue of the wing phenotypes and found a significant reduction in aberrant wing phenotypes when Magi was co-expressed with E6+UBE3A (Fig 5G). Consistent with this result, E6+UBE3A expressed in wing discs in a null mutant of Magi (Magi^bst^) [32] resulted in pupal lethality, suggesting an enhancement of the phenotype. To determine whether Magi-mediated rescue of E6+UBE3A was specific, we examined the rescue capability of other E6 PDZ target proteins including Dlg and Scrib; neither Dlg nor Scrib could suppress the effects of E6+UBE3A (Fig 5C, 5D and 5G). Examination of wing discs of third instar larvae revealed that overexpression of Magi completely blocked the apoptosis seen in E6+UBE3A co-expressing epithelia (n = 20 discs), whereas overexpression of Scrib did not (n = 10 discs) (Fig 5H–5M). These results suggest that Magi-mediated rescue of wing abnormalities is specific and that expression of Magi can block or reduce the defects caused by E6+UBE3A co-expression.

Next, we asked which domains of Magi were responsible for suppressing the E6+UBE3A mediated defects. *Drosophila* Magi contains four PDZ domains and two WW domains. We expressed Magi transgenes lacking either the two WW domains (MagiΔWW) or the PDZ domains (MagiΔPDZ) [34] in the wing disc, along with E6+UBE3A. Expression of MagiΔWW significantly rescued the wing abnormalities caused by E6+UBE3A (Fig 5F and 5G). Conversely expression of MagiΔPDZ failed to rescue the E6+UBE3A-mediated wing defects (Fig 5E and 5G). These results suggest an essential role for the PDZ domains of Magi in blocking the deleterious effects of E6+UBE3A. While Magi is unlikely to be the sole PDZ protein degraded by E6+UBE3A, our data suggests that Magi is an important degradation target and that the highly conserved PDZ domains play a critical role in targeting by HPV E6 and human UBE3A.

**Fig 6:**
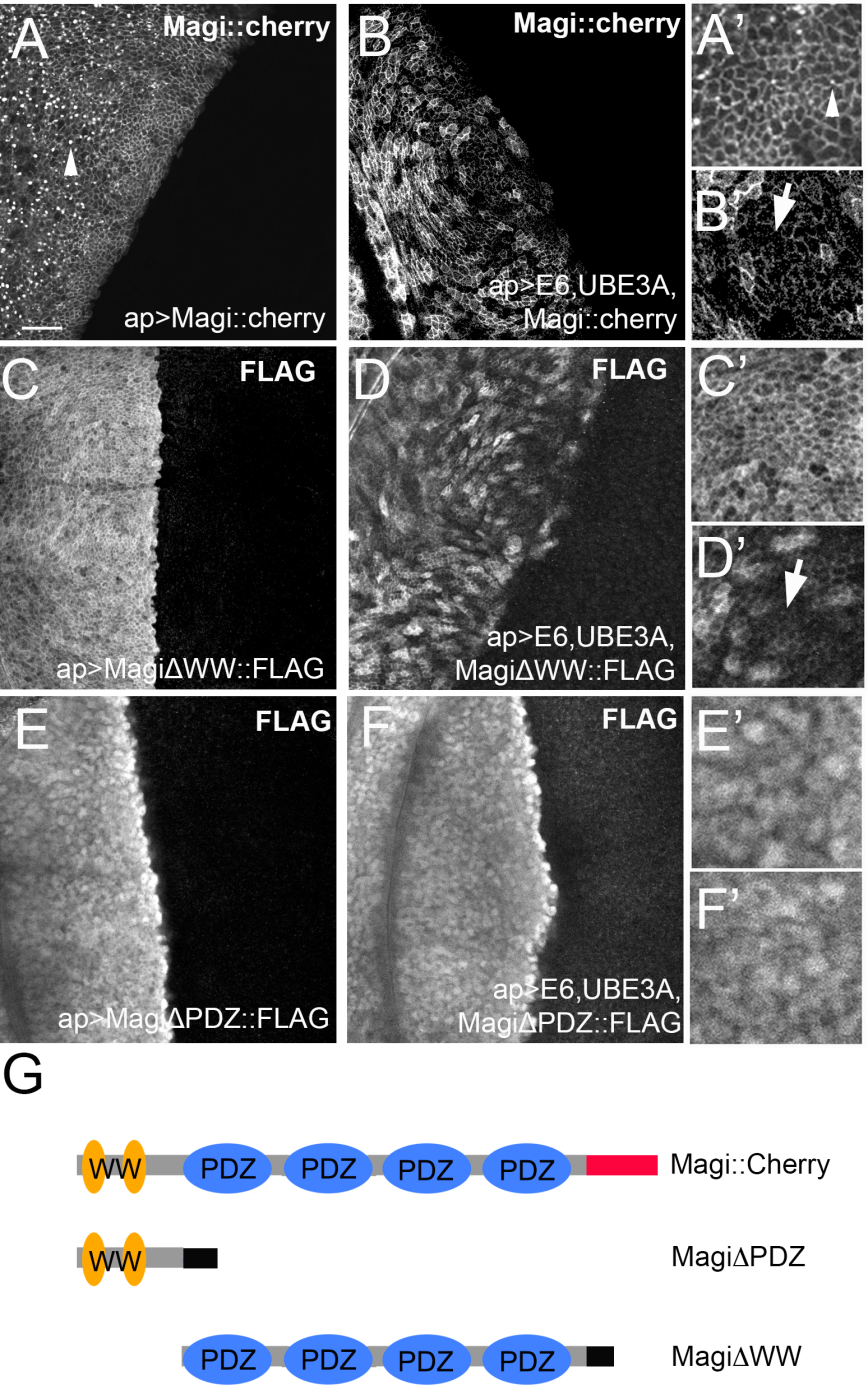
E6 targets the PDZ domains of Magi. Transgenes were expressed in the wing disc under the control of apterous-Gal4 driver in the dorsal compartment. Discs are oriented in all panels with dorsal to the left and ventral to the right. **(A-B)** Full length Magi::Cherry was distributed around the membrane and found in prominent intracellular puncta **(A,A’)**. When coexpressed with E6+UBE3A, Magi::Cherry levels in the membrane and puncta were reduced **(B,B’)** with regions of little or no expression (B’ arrow). **(C-D)** Magi∆WW is uniformly distributed around the membrane **(C,C’)**. When expressed with E6+UBE3A **(D,D’)** the levels of Magi∆WW are reduced with regions of little or no expression (D’ arrow). **(E, F)** The localization and levels of Magi∆PDZ were not affected in E6+UBE3A expressing compartment **(F,F’)** similar to Magi∆PDZ expression alone **(E,E’)**. **(G)** Cartoon of the Magi transgenic constructs with the WW (yellow), PDZ (blue) and epitope tags (Cherry–red; FLAG–black) indicated. Scale bars indicate 10μm. Inserts were digitally magnified 200%.

As neither Magi::Cherry nor MagiΔWW were able to fully rescue the defects caused by E6+UBE3A, we tested the degree to which each could be targeted for degradation by E6. We examined the levels and localization of Magi::Cherry as well as the MagiΔWW and MagiΔPDZ mutants in wing imaginal discs co-expressing E6+UBE3A. Using apterous-GAL4 to drive expression in the dorsal half of the disc, both Magi::Cherry and MagiΔWW proteins were reduced at the plasma membrane although this reduction was not uniform (Fig 6B and 6D). The Magi::Cherry expressed alone is uniformly distributed around the membrane and found in prominent intracellular puncta (Fig 6A), whilst in the presence of E6+UBE3A Magi::Cherry levels are reduced in the puncta and at the membrane. Conversely the localization and expression level of MagiΔPDZ was not affected when co-expressed with E6+UBE3A (Fig 6E and 6F). These results demonstrate that E6 was capable of degrading a proportion of the wild type Magi::Cherry and MagiΔWW proteins even when these were overexpressed, and this is likely the reason why a full rescue of the wing abnormalities was not obtained.

### HPV E6 in conjunction with oncogenic Ras and Notch causes tumorigenesis and malignancy

**Fig 7:**
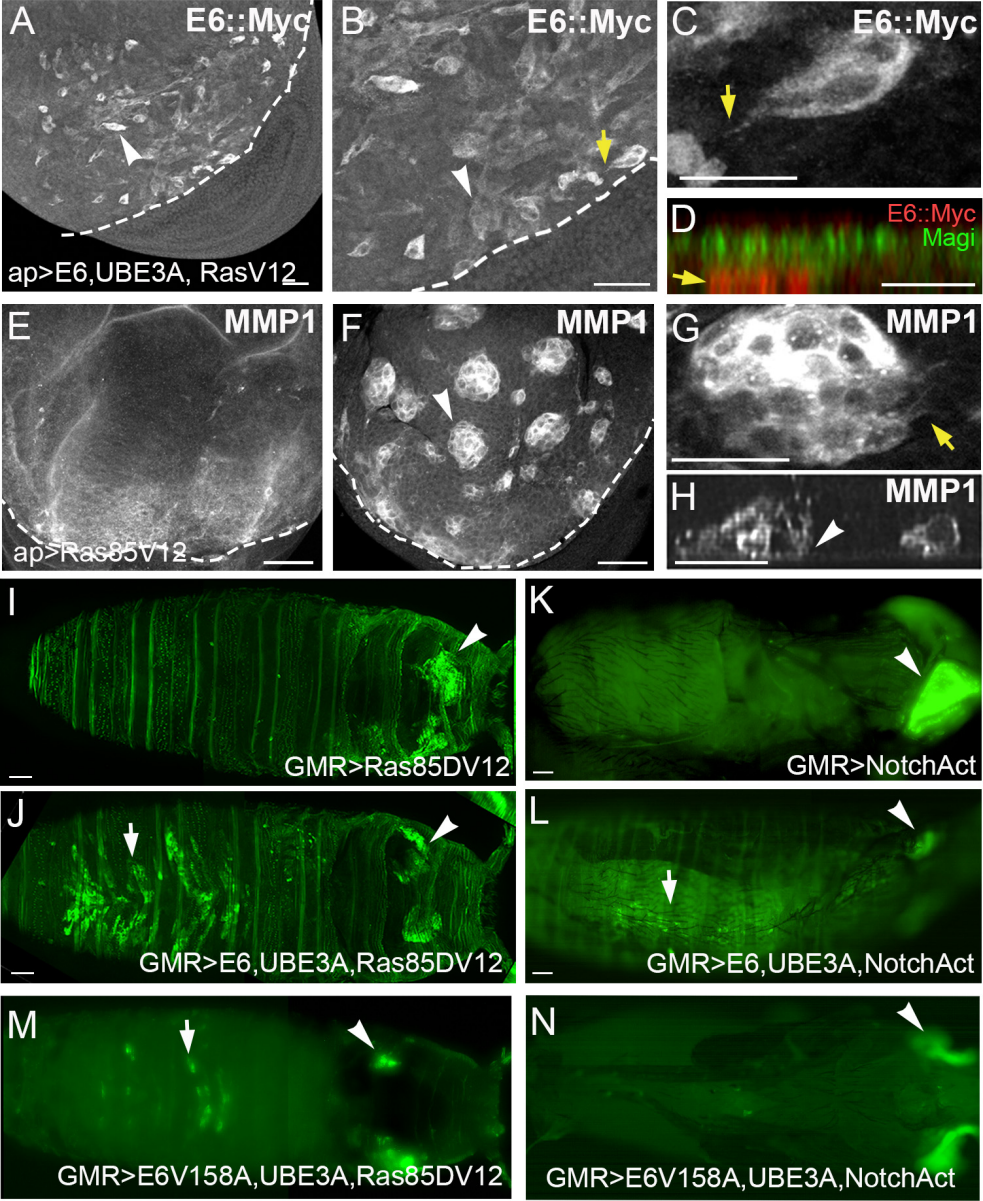
HPV E6 in conjunction with oncogenic Ras and Notch causes tumorigenesis and malignancy. **(A-H)** Transgenes were expressed under the control of apterous-Gal4 in the dorsal compartment of the wing disc. Dorsal is up and ventral is down in these panels. Dashed lines indicate the boundary between the dorsal and ventral compartments. **(A-D)** Expression of constitutively activated Ras with E6+UBE3A for 24 hrs in wing epithelia. **(A,B)** Cells positive for E6::myc displayed a flat and fibroblast-like morphology (arrowheads). **(B)** A higher magnification of A shows the expression is greater in single or small cell clusters. **(C)** Digital magnification of B shows cells displaying filopodial-like processes (arrow). **(D)** At 24 hours of expression, E6::Myc (red) labeled cells were found on the basal side of the epithelium (arrow), with the remaining Magi immunolabeling (green) marking the apical side. **(E)** Expression of constitutively activated Ras85DV12 (RasV12) alone. Over proliferation and the generation of extra folds in the tissues was observed but no mesenchymal-like cells or clusters of MMP1 expressing cells were seen. **(F-H)** Expression of Ras85DV12 with E6+UBE3A for 48 hrs. **(F)** Both individual and cell clusters that expressed high levels of MMP1 were observed and were limited to the apterous side. **(G)** Higher magnification of panel F showing the filopodial-like processes observed with many clusters (arrow). **(H)** A side projection of E6+UBE3A plus Ras85DV12 expressing disc. The MMP1 positive cell clusters are on the basal side of the epithelium (arrowhead). **(I-N)** Transgenes were expressed with the eye-specific driver, GMR-Gal4, and labeled with a membrane tagged GFP. Pupae **(I, J, M)** or pharate adults **(K, L, N)** are shown with anterior to the right and posterior to the left. **(I)** Activated Ras and GFP-positive cells do not migrate out of the developing eyes (arrowhead). **(J)** Expression of E6+UBE3A in the presence of Ras85DV12 resulted in migration, with many GFP-positive cells detected in the abdomen of these animals (arrow), distant from the source of E6+UBE3A+Ras85DV12 expression in the eye imaginal disc (arrowhead). **(K)** Activated Notch, NotchACT, and GFP positive cells do not migrate out of the developing eyes (arrowhead). **(L)** When E6+UBE3A were coexpressed with NotchACT, cells labeled with GFP were observed in the abdomen (arrow), far from the source of the expression (arrowhead). **(M)** Co-expression of E6V158A and UBE3A resulted reduced cell migration in the abdomen (arrow) away from the eye disc (arrowhead), compared with wildtype E6. **(N)** When E6V158A+UBE3A were co-expressed with Notch ACT, no GFP-labeled cells were observed in the abdomen away from the eye disc (arrowhead). Scale bars:A 20μm, B-H 10 μm, I-N 100 μm.

In humans there is usually a period of 15–20 years from the time of HPV infection to the development of cancer. Transgenic mice also showed a latency of 16–20 months for cancer development in presence of HPV E6 [61]. These results suggest that cooperation between E6 and UBE3A is insufficient to cause uncontrolled growth and malignant transformation of epithelia, and that genetic events, such as genomic instability and spontaneous mutation, may also play a role in E6-induced epithelial transformation. Our results are consistent with this view, as co-expression of E6+UBE3A was not sufficient to cause cellular transformation and cancer. Mutations in oncogenes such as Ras have been implicated in cancer progression and HPV-mediated tumorigenesis [61–63]. To investigate this in our model system we expressed E6+UBE3A in epithelial cells that expressed an activated Ras, Ras85DV12. As expression of Ras85DV12 driven by apterous-Gal4 is larval lethal, we carried out a temperature shift experiment where Gal4 was silenced during embryonic development using a temperature-sensitive Gal4 inhibitor, Gal80^ts^ [64,65]. A shift to 29°C during the second instar larval stage activated Gal4, and the wing imaginal discs of third instar larva after 24 hours at 29°C showed epithelial cells that expressed high levels of E6 and had altered morphology. These cells morphologically resembled mesenchymal cells (Fig 7A and 7B), in that they acquired a fibroblast-like, flat morphology (Fig 7B), they displayed filopodial-like processes (Fig 7C; arrow), and were delaminated at the basal side of the columnar epithelia (Fig 7D; arrow). However, only a subset of cells on the apterous side expressed high levels of E6::myc (Fig 7B). As we found the effects of E6 to be dosage-dependent, we increased the timing of expression of E6+UBE3A to 48 hours by shifting to 29°C during the first instar larval stage. Wing imaginal discs of third instar larva after 48 hours of expression had clusters and individual cells that expressed high levels of MMP1 (100% penetrant; n = 20 discs) (Fig 7F, arrow). Cell clusters also had extending filopodial-like processes (Fig 7G; arrow). Similar to when E6+UBE3A was co-expressed with p35 (Fig 4J’, 4M’ and 4P’), these clusters were found under the epithelium at the basal side (Fig 7H, arrow) and were limited to the apterous side of the disc with no spread into the wildtype, ventral compartment of the wing disc. Overexpression of Ras85DV12 in the absence of E6+UBE3A caused disc overgrowth as expected, but no mesenchymal-like cells or clusters expressing MMP1 were detected (Fig 7E). These results indicate that the combination of HPV E6 and UBE3A is insufficient to cause cellular transformation, which requires the additional cooperation of cellular oncogenes, such as activated Ras. It is also clear from this analysis that only a small proportion of E6-expressing cells underwent transformation, as initially we only saw single cells with altered morphologies, which, over time, expanded into clusters in a manner analogous to the clonal development of HPV-induced malignancies.

As HPV E6 has been implicated in the later stages of tumorigenesis, we wanted to determine whether coexpression of E6+UBE3A and Ras85DV12 could cause malignancy and metastasis. In order to do this we expressed E6+UBE3A, Ras85DV12 and a membrane-bound GFP marker (mCD8::GFP) using GMR-Gal4 in the eye, so that we could readily detect the migration of malignant cells into other regions of the body. Expression of E6+UBE3A in the presence of Ras85DV12 resulted in cellular migration, with many GFP-positive cells detected in the abdomen of these animals (Fig 7J, arrow), distant from the source of E6+UBE3A+Ras85DV12 expression in the eye imaginal disc (Fig 7J, arrowhead) (70% penetrant, n = 20 animals). GFP-positive cells were not detected in the abdomen when Ras85DV12 was expressed alone (Fig 7I) (n = 10 animals). Expression of Ras 85DV12 alone or in combination with E6 and UBE3A caused pupal lethality with extensive tissue necrosis making the analysis of metastasis in the adult impossible. Similar results were obtained when E6+UBE3A were co-expressed with an activated form of Notch (NotchACT) where E6+UBE3A+NotchACT was lethal at the pharate adult stage (prior to hatching). NotchACT plus E6+UBE3A cells labeled with GFP were observed in the abdomen (Fig 7L, arrow) distant from the eye imaginal disc (Fig 7L, arrowhead) (40% penetrant, n = 30 animals). GFP positive cells were never observed in the abdomen when NotchACT was expressed alone (Fig 7K) (n = 20 animals). These results indicate that E6 + UBE3A, when expressed with activated Ras or Notch, leads to many of the cellular phenotypes associated with EMT and spread of transformed cells throughout the body. In order to test if these phenotypes were dependent on the function of the PDZ binding motif of E6, we expressed the E6V158A mutant that is deficient in binding to PDZ proteins in conjunction with RaS85DV12 and UBE3A. In the absence of the PDZ protein interaction, we observed a reduced level of EMT and cell migration with a penetrance of 50% (n = 30 animals) (Fig 7M). Expression of E6V158A in the presence of activated Notch and UBE3A did not result in cell migration away from the eye disc (100% penetrant, n = 20) (Fig 7N). These results collectively suggest that E6 targeting of PDZ proteins plays a major role in EMT and cell migration induced by E6+UBE3A.

### A signaling pathway screen identified the insulin receptor as a downstream effector of E6

Given the high degree of conservation of its genes and signaling pathways, the *Drosophila* eye appeared to be an ideal model system to identify the signaling pathways involved in E6+UBE3A-mediated cell abnormalities. Therefore, we carried out a small genetic screen to identify which signaling molecules and pathways functionally interact with E6. We tested a range of signaling pathways (Table 1) and observed no enhancement or suppression of the E6+UBE3A phenotypes, with the exception of the insulin receptor. Interestingly, we found no interactions with pathways known to influence apoptosis, such as the JNK pathway, p53 and mutants in the mitochondrial apoptosis pathway (*Buffy* and *Debcl*) (S2 Fig), known to function downstream of the *Drosophila* retinoblastoma protein (pRb) [66]. In particular, blocking p53 function using a DN form [67] did not rescue the eye phenotypes (S2 Fig).

**Fig 8:**
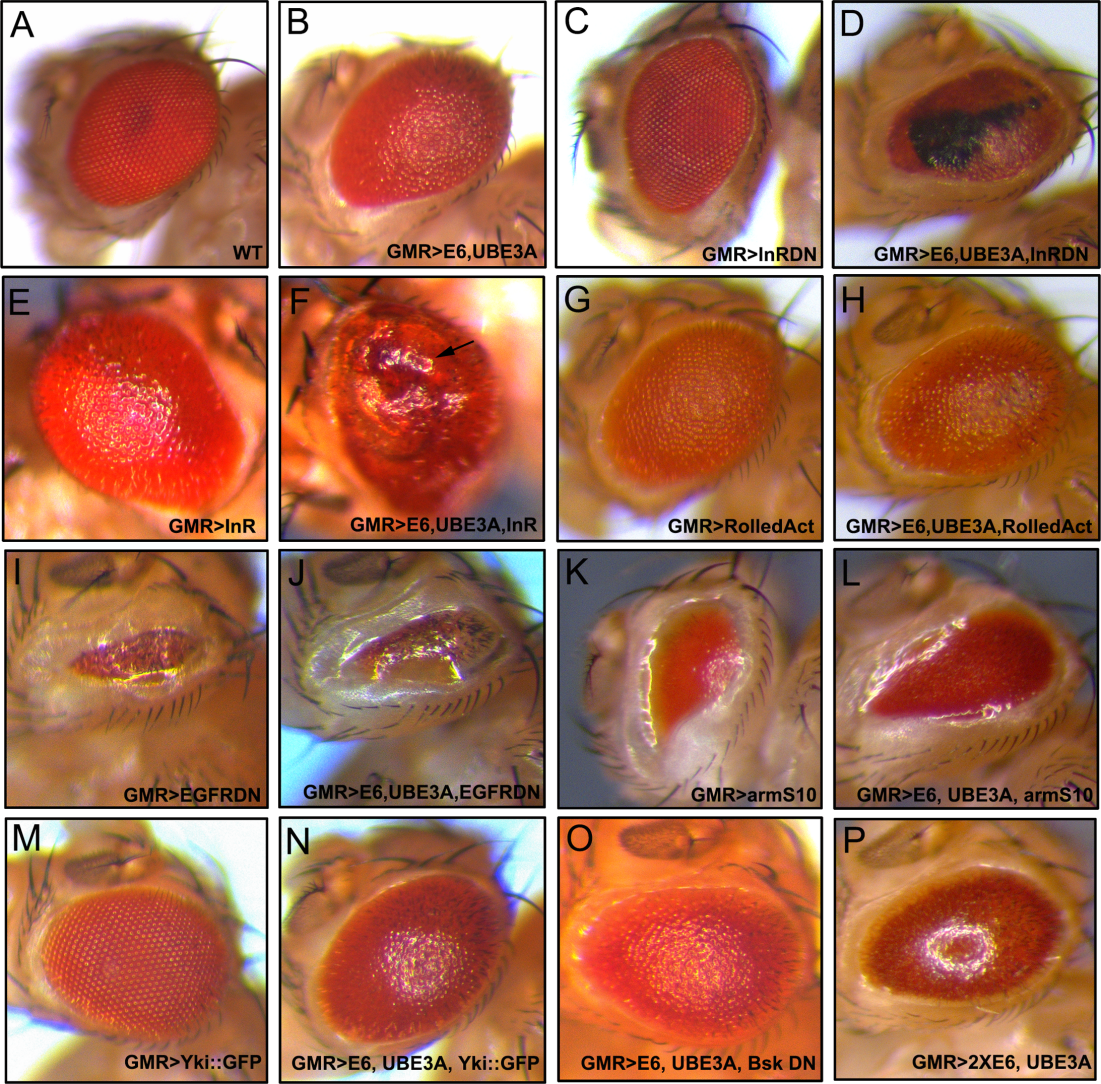
A signaling pathway screen identified the insulin receptor as an E6 modifier. Transgenes were driven with eye specific GMR-Gal4 driver. **(A)** Wild type eye. **(B)** E6+UBE3A expressing eye with a rough eye phenotype. **(C)** A dominant negative Insulin Receptor (InRDN) alone with eyes smaller than wild type. **(D)** A dominant negative InR (InRDN) together with E6+UBE3A caused necrosis (black regions). **(E)** Insulin receptor (InR) overexpression alone increased eye size. **(F)** Insulin Receptor (InR) together with E6+UBE3A triggered hyperplasia with out-pocketing of eye tissue. **(G)** Activated MAPK (Erk, *Drosophila* rolled) caused a rough eye. **(H)** Activated rolled, expressed simultaneously with E6+UBE3A, had no additional effect on the E6+UBE3A phenotypes. **(I)** A dominant negative form of EGFR (EGFR DN) alone caused very small eye phenotype. **(J)** Dominant negative EGFR (EGFR DN) together with E6+UBE3A had no additional effect on E6+UBE3A phenotypes. **(K)** Activated beta-catenin (*Drosophila* armadillo (armS10)) resulted in small eye phenotype. **(L)** Activated arm (armS10) together with E6+UBE3A, had no additional effect on E6+UBE3A. **(M)** The downstream effector of Hippo signaling, Yki::GFP had no eye phenotype. **(N)** Yki::GFP together with E6+UBE3A neither enhanced nor suppressed the E6+UBE3A phenotype. **(O)** A dominant negative JNK, (*Drosophila* Bsk DN) together with E6+UBE3A had no additional effect on the E6+UBE3A eye phenotype. **(P)** Two copies of the E6 transgene plus UBE3A enhanced the defects caused by E6+UBE3A expression. Eyes are smaller and glossy compared to eyes with one copy of E6 plus UBE3A (compare with B).

On the other hand, when we blocked insulin signaling by expressing a dominant negative form of the insulin receptor (InRDN) in conjunction with E6+UBE3A, the resulting eyes displayed large necrotic scars (100% of eyes, n = 70), which is an indication of cell death (Fig 8D). Co-expression of the insulin receptor (InR) in E6+UBE3A-expressing eyes resulted in hyperplasia (Fig 8F; 100% of eyes, n = 100) compared with the expression of insulin receptor alone, which generated bigger eyes but no hyperplasia (Fig 8E). In contrast, changes to the EGF receptor (EGFR) (Fig 8J), or the MAP kinases ERK (*Drosophila* Rolled) (Fig 8H) and JNK (*Drosophila* Basket) (Fig 8O), had no effect on the E6+UBE3A eye phenotypes. Similarly, disruption of Wnt signaling with activated beta-catenin (*Drosophila* Armadillo) (Fig 8L) or Hippo signaling (*Drosophila* Yorkie) (Fig 8N) had no effect. Thus, none of the other pathways tested showed any effect upon the E6+UBE3A phenotype, indicating that the effect of the insulin receptor is specific and suggesting that changes in insulin signaling may play a role in in the cell transformation and cancer progression induced by HPV.

## Discussion

In this study we have developed a new model of E6+UBE3A-mediated cell transformation in *Drosophila melanogaster*, an excellent system for further investigating the molecular mechanisms underlying E6-mediated cellular transformation. In our model we found that E6 expression alone is insufficient to disrupt the host cellular function but dysfunction also requires the human E3 ubiquitin ligase, UBE3A [20,21]. These results suggest that *Drosophila* UBE3A does not interact with the E6 protein, and this would be expected as *Drosophila* UBE3A lacks the critical interaction site which is bound by E6 on human UBE3A [68]. In support of this, co-expression of E6 and human UBE3A in the eye and wing resulted in severe defects. E6+UBE3A were insufficient alone to induce cellular transformation. When E6+UBE3A expression was combined with expression of activated Ras or Notch, it resulted in cellular transformation, EMT and cell migration throughout the body. This is consistent with previous findings in transgenic mouse models where activated Ras, in combination with E6, results in the formation of malignant tumors [62]. Similarly, mutations in the Ras oncogene have been reported in HPV tumors [61,63]. Our results are consistent with these results, and together they support the notion that HPV alone is insufficient to generate malignant tumors and a second hit or mutation in a cellular oncogene is necessary. The expression of the HPV E7 protein in our model would be of interest to determine if the presence of E7 could increase the degree of cell transformation or allow cell transformation to occur at earlier stages in the life cycle.

We further show that E6 targets a number of PDZ domain-containing proteins in *Drosophila* with apparently different degrees of degradation. Magi is a major target of E6, showing a markedly greater susceptibility to E6-induced degradation compared with other polarity proteins such as Dlg and Scrib. Previous studies have also identified MAGI-1 as a major target of high-risk human papillomaviruses HPV 16 and 18 E6 oncoproteins in vertebrate epithelial cells [15]. We found that *Drosophila* Magi is also highly susceptible to E6-induced degradation *in vitro*, in a manner analogous to that of MAGI-1.These findings suggest that there is a hierarchy of E6 targets in flies, and indicates that a number of cellular pathways targeted by E6 are conserved between insects and mammals. Interestingly, we did not observe any degradation of P53 in the presence of E6+UBE3A and an expression of a dominant negative form of P53 in E6+UBE3A expressing eyes did not have any effect on the cellular defects caused by E6+UBE3A co-expression. P53 is a key target of HPV E6-mediated polyubiquitination and degradation [69]. Our results collectively suggest that the cellular defects caused by E6+UBE3A co-expression in *Drosophila* are independent of P53 degradation, making our model system potentially useful for investigating the other activities of E6+UBE3A on cellular transformation.

The degradation of Magi and the other PDZ domain-containing proteins requires an intact E6 PBM, demonstrating that the mechanism of interaction between E6 and its PDZ containing targets is conserved between insects and mammals. The cellular defects caused by E6+UBE3A co-expression could be partly due to loss or disruption of a suite of PDZ domain proteins targeted by E6. Both Dlg and Scrib are polarity proteins and a reduction of these regulators, or a disruption in the balance between the levels of these proteins, could affect the polarity of cells as suggested by the mislocalization of polarity protein Baz (Par3) in the eye. Magi plays a role in remodeling adherens junctions in interommatidial cells in the eye, and loss of Magi disrupts the organization of the interommatidial cells [70]. Consistent with this we saw disorganization of interommatidial cells and mislocalization of the adherens junction protein Armadillo (beta-catenin), suggesting that loss of Magi may play a role in the development of E6+UBE3A-mediated cellular abnormalities. In support of this, overexpression of a full-length Magi, or a transgene lacking the two WW domains, partially rescued the E6+UBE3A-mediated defects, suggesting that disruption of Magi function was in part responsible for the observed phenotypes. Interestingly, a mutant Magi lacking the PDZ domains was unable to rescue the E6+UBE3A-induced defects, suggesting that a function of Magi involving PDZ interactions is essential for this activity, and that this is the function of Magi that is targeted by E6 and UBE3A. The incompleteness of the rescue obtained with wild type Magi could be for a number of reasons, including E6 degradation of the overexpressed Magi, or the possibility that other cellular targets that are degraded by E6 also play an important role. Indeed, many other PDZ proteins have been reported to be targets of HPV E6 including PSD95 [71], PATJ [72,73], MUPP1 [16], TIP1 [74], TIP2 [75], PTPN3 [76,77], PTPN13 [78], and CAL [79]. A number of these proteins have homologs in *Drosophila* and are involved in important cellular processes including cell-cell junction, cell polarity and regulation of cell signaling. Hence disruption of these in combination could play a role in E6+UBE3A-mediated cellular defects. Using our model system we are in a position to determine the hierarchy and importance of targeting these PDZ proteins in HPV E6-mediated cellular transformation.

Identifying Magi as a major target of HPV E6 in our model is similar to what is observed in human cells, and supports the finding that Magi has a significant biological relevance to HPV infection. Expression of a form of MAGI-1 resistant to E6-mediated degradation represses cancer cell proliferation and induces apoptosis [80]. Loss of tight junction integrity in an HPV-positive, tumor-derived cell line results from E6-mediated degradation of MAGI-1 [15]. These studies suggest that MAGI-1 is a key factor in cellular function and that its removal can contribute to cellular transformation. Strikingly, loss of the sole *Magi* gene in *Drosophila* has no deleterious effect, and animals deficient for Magi live to adulthood with no detectable abnormalities [39,70]. However, this does not rule out the importance of Magi as an important target of E6, as *Drosophila* mutants of another major HPV target, p53, are also adult viable. *p53* mutants (in both mouse and *Drosophila*) live to adulthood with no detectable abnormalities [81,82]. However, these animals are more prone to developing cancer, and when exposed to stress-inducing conditions and genomic instability, such as irradiation, they fail to trigger apoptotic cell death to remove the damaged cells [82–86]. Hence, it is plausible that Magi could possess some tumor suppressor activity. Indeed, Magi, like p53, is an extremely susceptible target for E6-mediated degradation, and therefore is unlikely to be subject to any selection pressure to mutate during the development of HPV-induced malignancy. Therefore, it is not surprising to find that, like p53, it is wild-type in most cervical cancers, with MAGI-1 mutations detected in less than 1% of cervical cancers tested (COSMIC, Catalogue of Somatic Mutations in Cancers: http://cancer.sanger.ac.uk/cosmic/gene/analysis?ln=MAGI1_ENST00000402939). Mutations, or aberrations in expression, of MAGI family members, have been found in global analyses of a number of different human cancers [87–92]. However it is not yet clear whether these mutations are drivers of carcinogenesis, or passengers.

As the molecular mechanism and the steps that lead to tumor formation and malignancy in HPV-positive cells are largely unknown, establishing a model of E6-mediated cell transformation that is amenable to large scale, unbiased genetic analysis is essential. Our *Drosophila* model of HPV E6-mediated cell transformation is highly amenable to genetic manipulation, with a strong degree of conservation at the gene and cellular levels. As a first test of this model we have identified the insulin receptor as a potential partner in the cellular transformation mediated by expression of E6+UBE3A, using the classic approach of testing for genetic interaction in the *Drosophila* eye. A growing number of studies have implicated the insulin receptor pathway in cancer development and progression. Conditions associated with insulin resistance such as Type 2 diabetes mellitus (T2DM), obesity, and metabolic syndrome are now recognized as major risk factors for development and progression of cancer [93–98]. Increased levels of insulin receptor have been reported in cancer cells [99,100] and insulin-like growth factor (IGF) signaling was shown to play a role in HPV-infected lesions and tumorigenesis [101]. Additionally, E6 can cause hyper-activation of the insulin receptor and activation of downstream signaling, including the PI3K and MAPK pathways [102]. For instance, the ability of HPV E6 plus E7 to transform keratinocytes is increased after down-regulation of the insulin-like growth factor binding protein 2 (IGFBP2) [101]. IGFBP2 in this system suppresses Insulin Growth Factor (IGFI/II), such that when IGFBP2 is down regulated IGFI/II stimulates the IGF receptor 1 (IGF1R) and AKT signaling. In high-grade pre-malignant cervical lesions infected with HPV16 the IGFBP2 levels are reduced, suggesting that changes in insulin signaling may play a key role cancer progression [101]. Earlier studies also revealed that E6 binds and degrades TSC2, a component of insulin signaling necessary for blocking the mTOR complex and growth, thus leading to activation of mTOR signaling and induction of growth [103]. These findings, together with our results, indicate that insulin signaling plays a critical role in HPV-mediated carcinogenesis and hence it is of significant interest to further explore the underlying mechanism of hyperplasia caused by interaction between HPV E6 and the Insulin receptor identified in this study.

Altogether we believe that our *Drosophila* E6+UBE3A model is an important new tool to study the molecular mechanism underlying HPV-mediated cancer and malignancy. As adult flies expressing E6+UBE3A exhibit severe wing and eye abnormalities this represents a uniquely valuable model with which to conduct modifier genetic screens to identify molecules that can inhibit E6-induced cellular defects. As a proof of principle, we have confirmed Magi as an essential element in the ability of E6 to induce cell transformation, demonstrated cooperation between E6 and different cellular oncogenic signaling pathways, and identified the insulin receptor as a downstream effector of E6 induced malignancy.

## Supporting Information

S1 Fig E6 alone does not trigger degradation and E6+UBE3A does not target Baz or Ecad. UAS-transgenes were expressed under the control of apterous-Gal4 driver in the dorsal compartment of the wing imaginal discs. In all images dorsal is to the left and ventral is to the right. Dashed lines indicate the boundary between the dorsal and ventral compartments. **(A-C)** Co-expression of E6 and UBE3A had no effect on PDZ protein Bazooka (Baz/Par-3). **(E-G)** Co-expression of E6 and UBE3A had no effect on the PDZ domain protein Par-6 **(I-K)** Co-expression of E6 and UBE3A had no effect on the adherens junction protein Ecad. **(M-O)** Expression of E6 alone had no effect on the level or localization of Magi. **(Q-S)** Expression of E6 alone had no effect on the level or localization of Dlg. **(U-W)** Expression of human UBE3A alone had no effect on the levels or localization of Magi or Dlg. **(Y, Z)** Co-expression of E6V158A and UBE3A had no effect on the level of Dlg **(Y)** or Scrib **(Z)**. **(D, H, L, P, T, X, Y’, Z’)** Graphs representing the quantification results for the levels of Baz, Ecad, Magi, Dlg, and Magi respectively. n = 5 for each experiment. ns indicates that the difference is not statistically significant. Error bars indicate SEM. Scale bars indicate 10μm.
(TIF)

S2 Fig E6+UBE3A-mediated defects are not mediated by other targets of E6 including regulators of apoptotic cell death and P53. Transgenes were driven with eye specific GMR-Gal4 driver. **(A)** E6+UBE3A expressing eye with a rough eye phenotype. Reduction of *Buffy* **(B)**, *Debcl* **(C)** or overexpression of Debcl **(D)** had no effect on the E6+UBE3A mediated eye defects. **(E)** Expression of a dominant negative form of P53 (P53 H159N) had no effect on the E6+UBE3A eye phenotype. **(F)** Control eye for D in which overexpression of Debcl has a similar effect on the eye as when Debcl is co-expressed with E6+UBE3A.
(TIF)
