# Supplementary figures and images for: A Drosophila Model of HPV E6-Induced Malignancy Reveals Essential Roles for Magi and the Insulin Receptor
Source: PLoS Pathog. 2016 Aug 18;12(8):e1005789. doi: 10.1371/journal.ppat.1005789 (PMC4990329; doi:10.1371/journal.ppat.1005789)

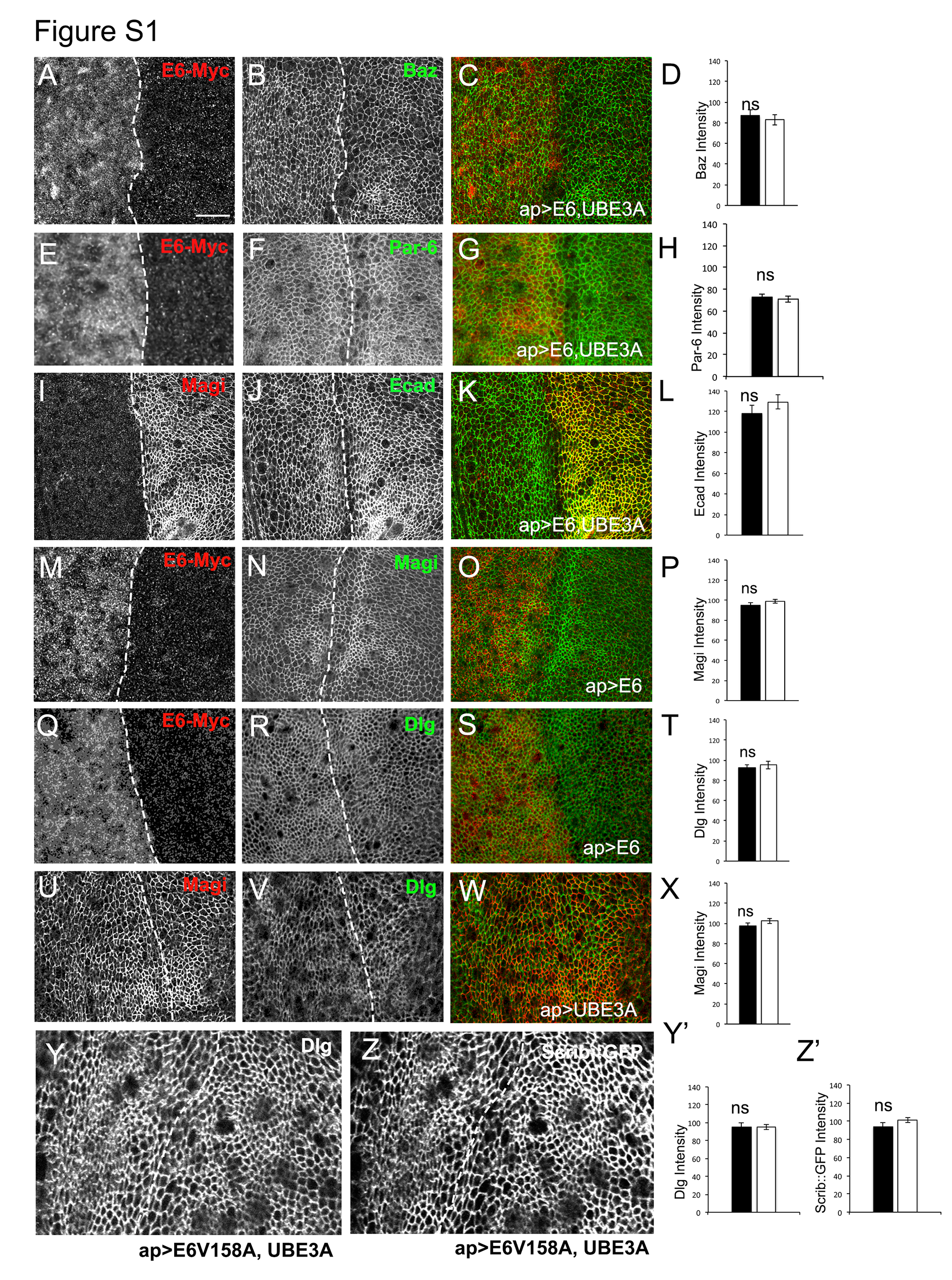

Supplement: S1 Fig — UAS-transgenes were expressed under the control of apterous-Gal4 driver in the dorsal compartment of the wing imaginal discs. In all images dorsal is to the left and ventral is to the right. Dashed lines indicate the boundary between the dorsal and ventral compartments. (A-C) Co-expression of E6 and UBE3A had no effect on PDZ protein Bazooka (Baz/Par-3). (E-G) Co-expression of E6 and UBE3A had no effect on the PDZ domain protein Par-6 (I-K) Co-expression of E6 and UBE3A had no effect on the adherens junction protein Ecad. (M-O) Expression of E6 alone had no effect on the level or localization of Magi. (Q-S) Expression of E6 alone had no effect on the level or localization of Dlg. (U-W) Expression of human UBE3A alone had no effect on the levels or localization of Magi or Dlg. (Y, Z) Co-expression of E6V158A and UBE3A had no effect on the level of Dlg (Y) or Scrib (Z). (D, H, L, P, T, X, Y’, Z’) Graphs representing the quantification results for the levels of Baz, Ecad, Magi, Dlg, and Magi respectively. n = 5 for each experiment. ns indicates that the difference is not statistically significant. Error bars indicate SEM. Scale bars indicate 10μm. (TIF) [file ppat.1005789.s001.tif]

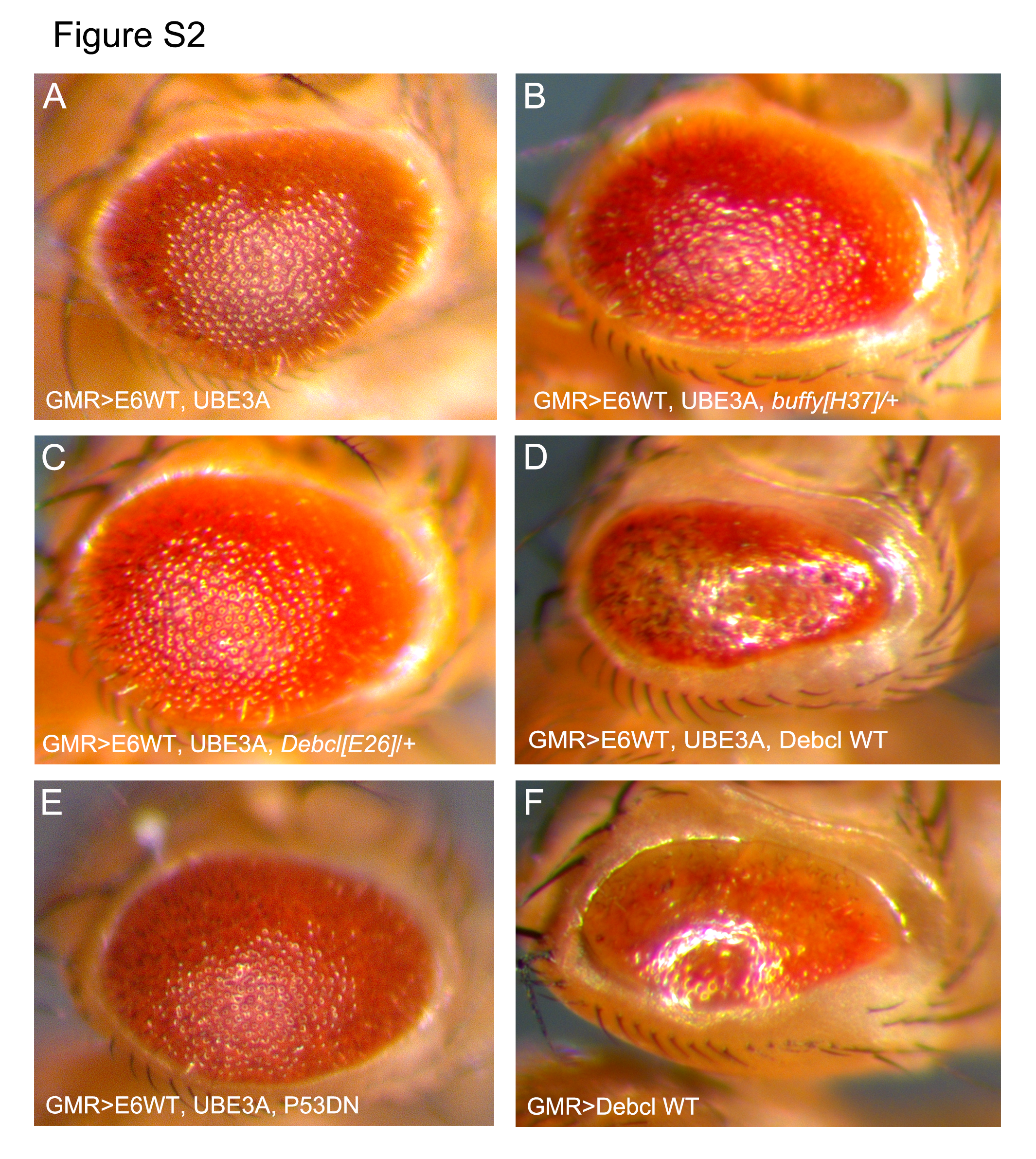

Supplement: S2 Fig — Transgenes were driven with eye specific GMR-Gal4 driver. (A) E6+UBE3A expressing eye with a rough eye phenotype. Reduction of Buffy (B), Debcl (C) or overexpression of Debcl (D) had no effect on the E6+UBE3A mediated eye defects. (E) Expression of a dominant negative form of P53 (P53 H159N) had no effect on the E6+UBE3A eye phenotype. (F) Control eye for D in which overexpression of Debcl has a similar effect on the eye as when Debcl is co-expressed with E6+UBE3A. (TIF) [file ppat.1005789.s002.tif]
